# Diagnostic challenge: pancreatic cancer masked by peripancreatic fluid collection after acute pancreatitis

**DOI:** 10.1007/s12328-025-02094-2

**Published:** 2025-01-08

**Authors:** Akram Ahmad, Zaid Ansari, Marah Karablieh, Osama Sherjeel Khan, Tilak Shah

**Affiliations:** Department of Gastroenterology, Cleveland Clinic Florida, Weston, FL 33326 USA

**Keywords:** Pancrease cyst, Pancerase cancer, EUS

## Abstract

Pancreatic cancer (PC) manifests as a highly aggressive neoplastic growth, ranking as the fourth major contributor to cancer-related mortality in the United States. Despite sustained efforts, the incidence of PC is projected to rise, and the mortality rate has seen only a marginal reduction over time. A mere 15% of pancreatic cancer cases are deemed resectable upon presentation, explaining the notably low 5-year survival rate associated with this malignancy. Acute pancreatitis (AP) encompasses various degrees of inflammation in the pancreas, leading to diverse outcomes. While commonly associated with gallstone and alcohol use, it can serve as the initial presentation of PC in approximately 1% of cases. Our case series highlights two patients diagnosed with pancreatic cancer (PC) following an episode of acute pancreatitis (AP). It is not uncommon for PC to be preceded by AP, with up to 5.9% of PC cases in the United States presenting similarly.

## Introduction

Pancreatic cancer (PC) manifests as a highly aggressive neoplastic growth, ranking as the fourth major contributor to cancer-related mortality in the United States [1]. Despite sustained efforts, the incidence of PC is projected to rise, and the mortality rate has seen only a marginal reduction over time [2, 3]. This can be attributed to the indolent nature of the disease, resulting in delayed detection. A mere 15% of pancreatic cancer cases are deemed resectable upon presentation, explaining the notably low 5-year survival rate associated with this malignancy [3].

Acute pancreatitis (AP) encompasses various degrees of inflammation in the pancreas, leading to diverse outcomes. While commonly associated with gallstone and alcohol use, it can serve as the initial presentation of PC in approximately 1% of cases [4]. Pancreatic ductal adenocarcinoma (PDAC) can present as acute pancreatitis, often due to the obstruction of the pancreatic duct by the tumor. This obstruction can lead to upstream ductal hypertension and subsequent pancreatitis [5]. This percentage rises within the population at risk and when the pancreatitis etiology is unidentified. Generally, a diagnosis of PC following AP offers a favorable prognosis due to the earlier detection of PC, often occurring a few months after the pancreatitis episode [6]. However, this detection can be delayed, extending to many years. In our paper, we present two cases of PC where the diagnosis was obscured by walled-off necrosis following an episode of acute pancreatitis.

## Case presentation

### Case 1

**Fig. 1:**
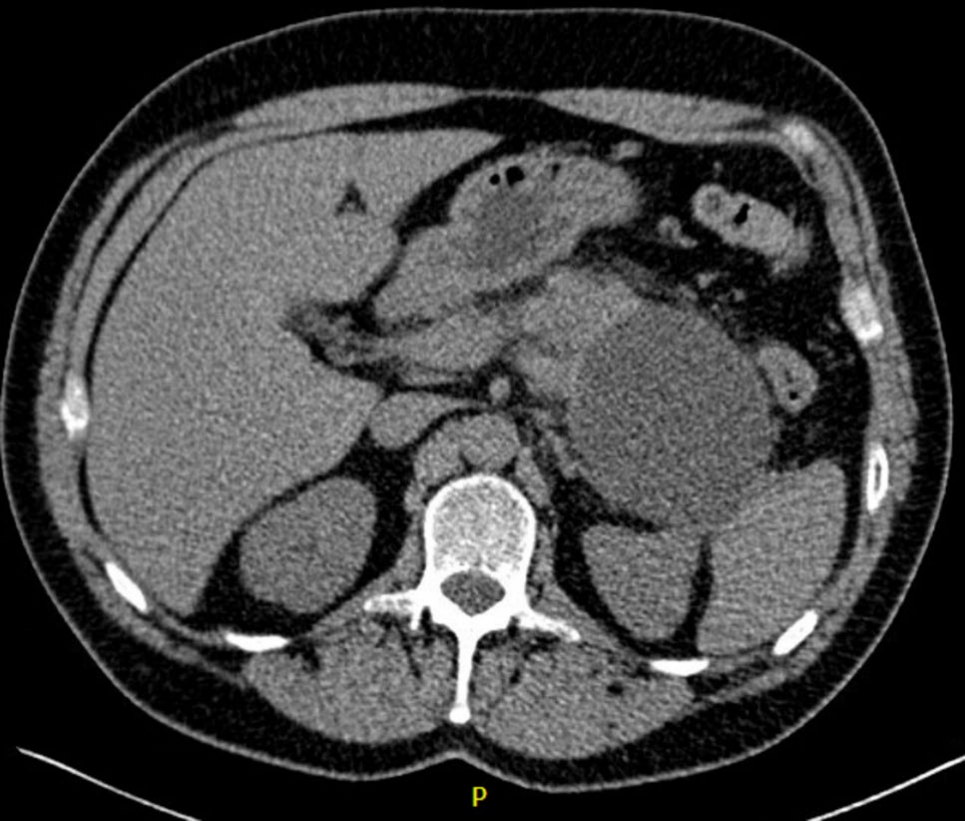
A 8.8 cm cyst with a mature wall in the body and tail of the pancreas

**Fig. 2:**
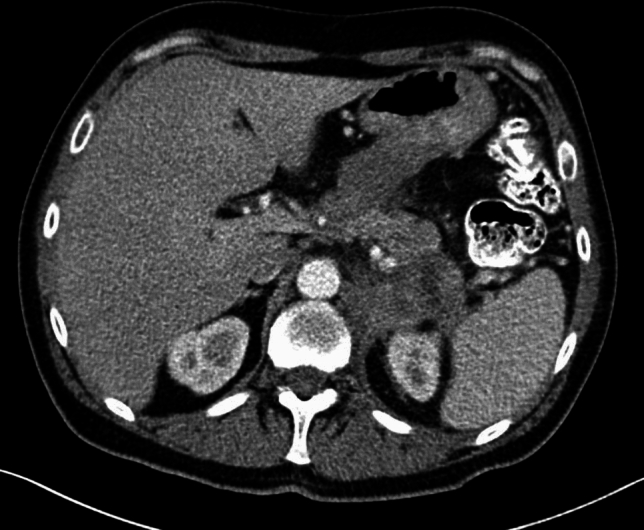
CT showing pancreatic mass taken at time of diagnosis of pancreatic cancer

**Fig. 3:**
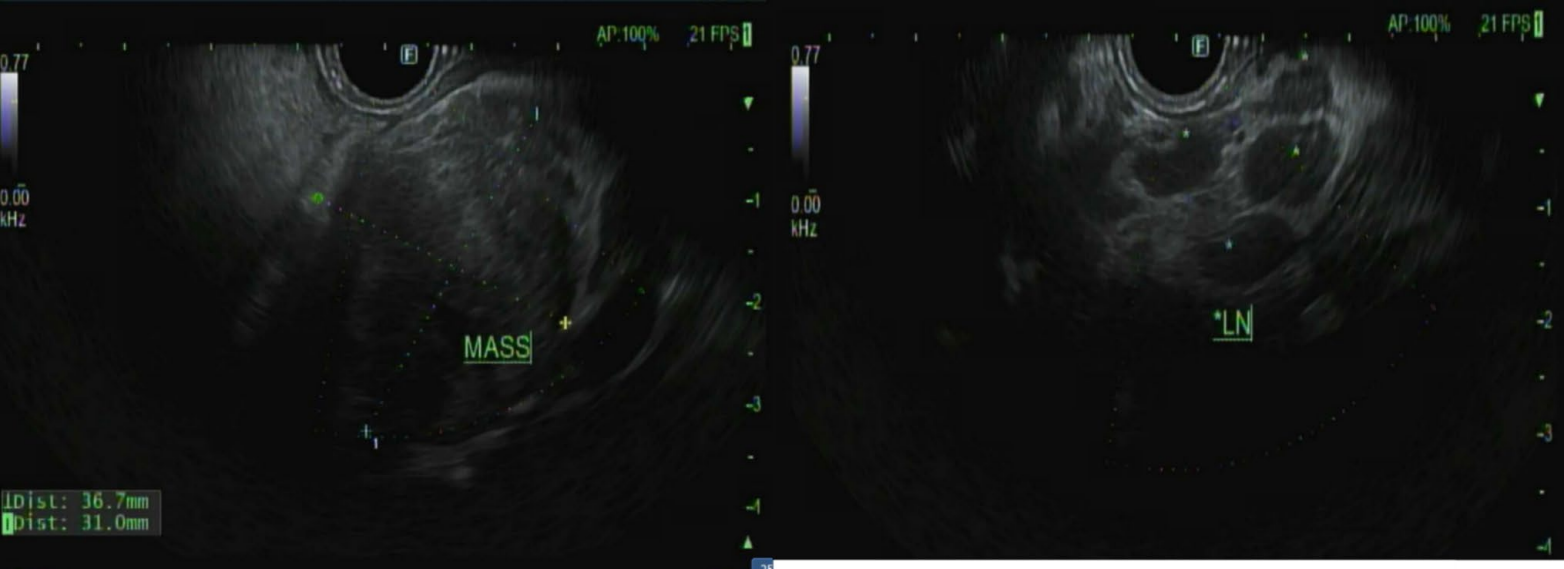
EUS demonstrating an irregular hypoechoic mass measuring 37 mm by 31 mm in cross-section in the pancreatic body

**Fig. 4:**
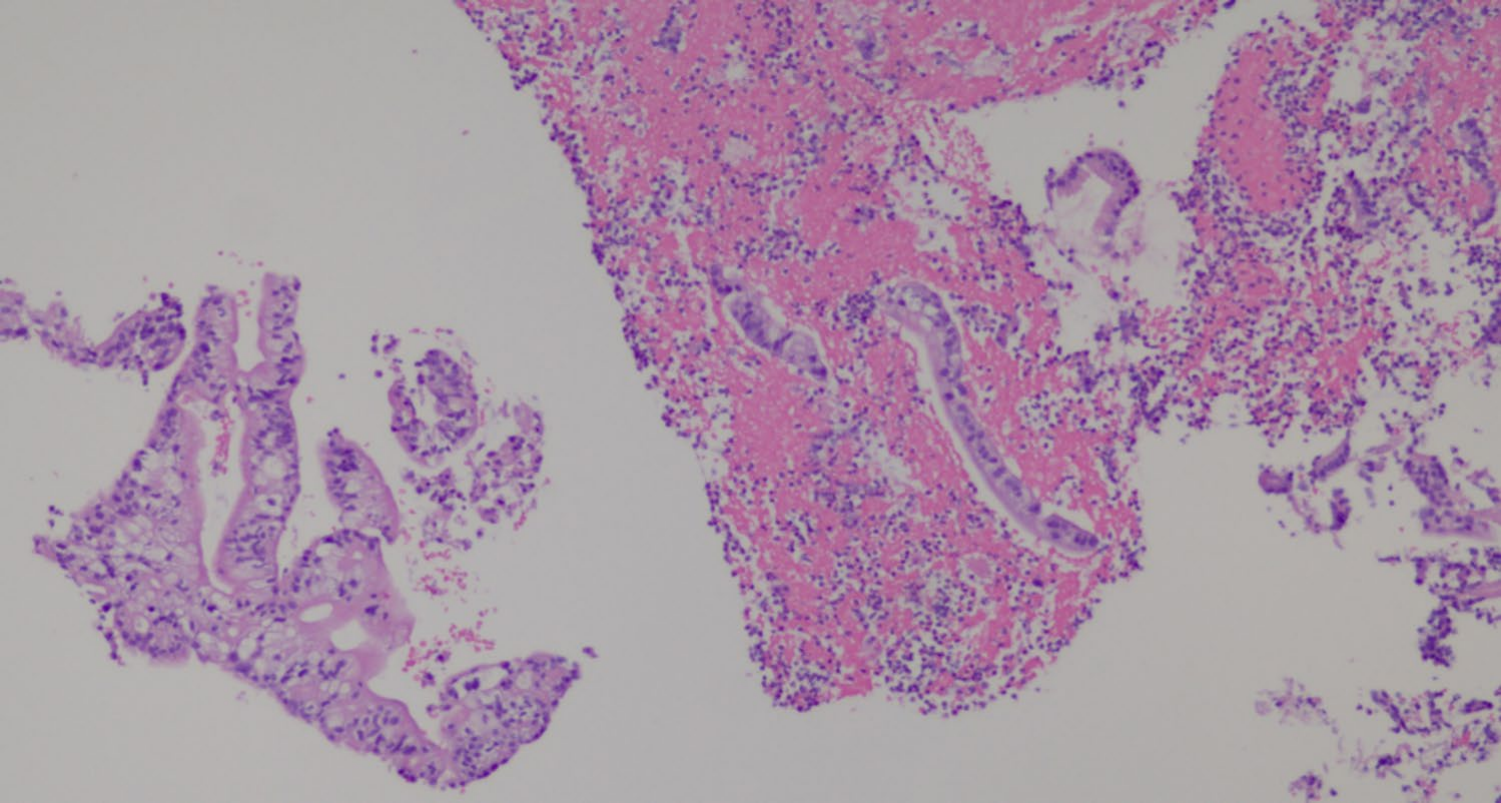
Pancreas FNA H&E 20x, histopathology of the FNA biopsy indicating adenocarcinoma

A 53-year-old male patient with a history of heavy alcohol and tobacco use was evaluated for constant epigastric discomfort 2 weeks after a presumed episode of acute pancreatitis. He reported a history of recurrent acute pancreatitis. Computed tomography (CT) scan of the abdomen without contrast identified an 8.8 cm cyst with a mature wall in the body and tail of the pancreas (Fig. 1). Given the characteristic clinical and imaging findings for a pseudocyst, the patient underwent endoscopic ultrasound (EUS)-guided cyst-gastrostomy using a lumen-apposing metal stent (LAMS). The appearance was suggestive of a pseudocyst; there was no tumor parenchyma seen around the cyst; contrast-enhanced EUS is not commonly done in the United States and, therefore, was not performed. Both the EUS and CT imaging done did not demonstrate any evidence of communication between the cyst and pancreatic duct. Cyst fluid analysis of thin brown chocolate-colored fluid showed the following: Amylase 47,672 U/L and Carcinoembryonic antigen (CEA) 5697 ng/mL. Given the markedly elevated cyst fluid CEA, a biopsy of the cyst wall was performed after inserting the gastroscope directly into the cyst cavity before LAMS removal. Biopsy showed fibrinopurulent exudate and fibrous tissue. The patient had clinically improved, so after a discussion at the multidisciplinary pancreas tumor board, a decision was made to observe the patient. CT with contrast or MRCP was not perused at that time as the clinical and imaging characteristics were typical of acute pancreatitis/associated pseudocyst. Over the next several months, the patient had four additional CT scans of the pancreas with intravenous (IV) contrast. These showed continued improvement in fluid collections, improved peripancreatic inflammatory changes, and a decrease in retroperitoneal adenopathy. However, the patient continued to experience epigastric pain and unintentional weight loss, so tumor markers were obtained and were as follows: CEA 7.4 ng/mL and CA19-9 of 3190 U/mL. A CT of the abdomen done at the time demonstrated a mass with ill-defined tissue density (Fig. 2). Subsequently, EUS was repeated, which identified an irregular hypoechoic mass measuring 37 mm by 31 mm in cross-section in the pancreatic body (Fig. 3). A fine needle biopsy was performed, which confirmed the mass was an adenocarcinoma (Fig. 4). Staging done for the tumor showed a tumor stage 3 (locally advanced). The patient was referred to oncology, and received FOLFIRINOX regimen followed by distal pancreatectomy, splenectomy, cholecystectomy, distal gastrectomy with RY gastrojejunostomy; adjuvant chemotherapy with gemcitabine/paclitaxel; course complicated by pulmonary embolism, chronic pain; and the patient subsequently opted for hospice care services.

### Case 2

**Fig. 5:**
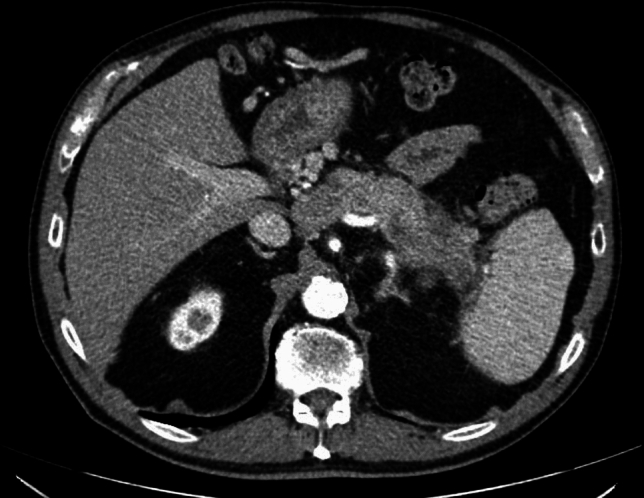
CT demonstrating surrounding inflammatory fat stranding around pancreas consistent with acute pancreatitis

**Fig. 6:**
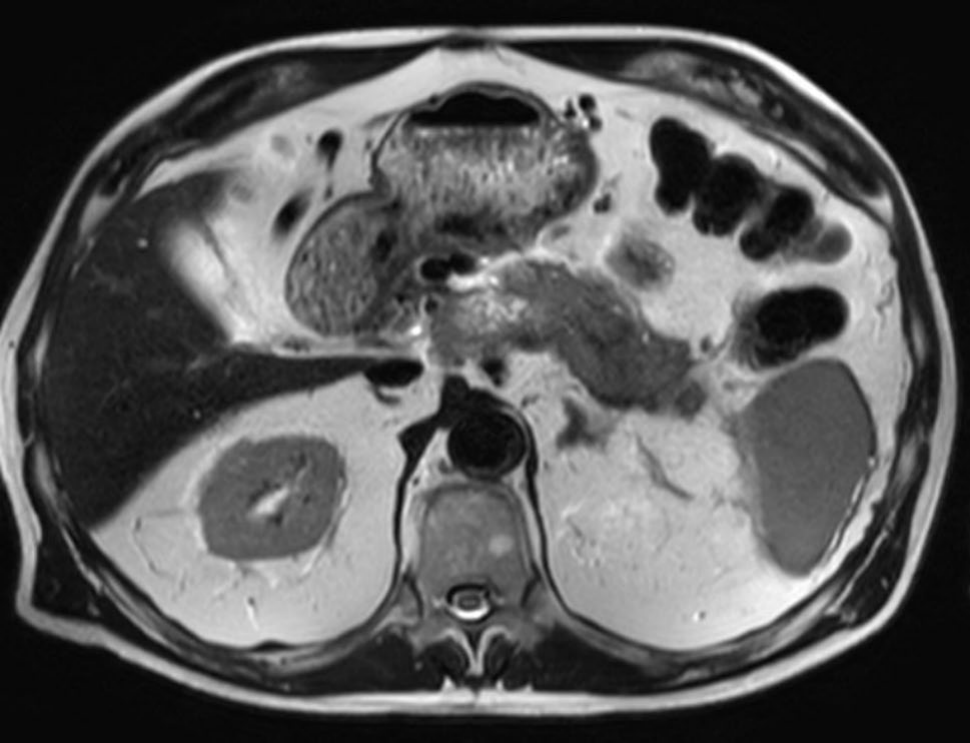
MRI done 2 weeks after initial CT scan, axial T2 sequence. Extensive peripancreatic phlegmonous changes with fluid extending toward the splenic hilum

**Fig. 7:**
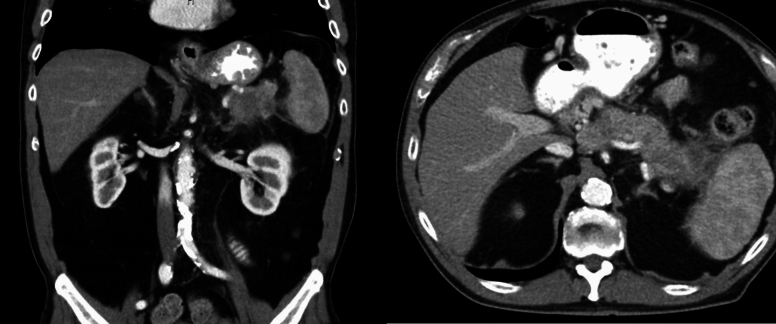
CT done 2 months later showing interval progression in phlegmonous changes around pancreatic tail and body with increase in surrounding inflammatory fat stranding

**Fig. 8:**
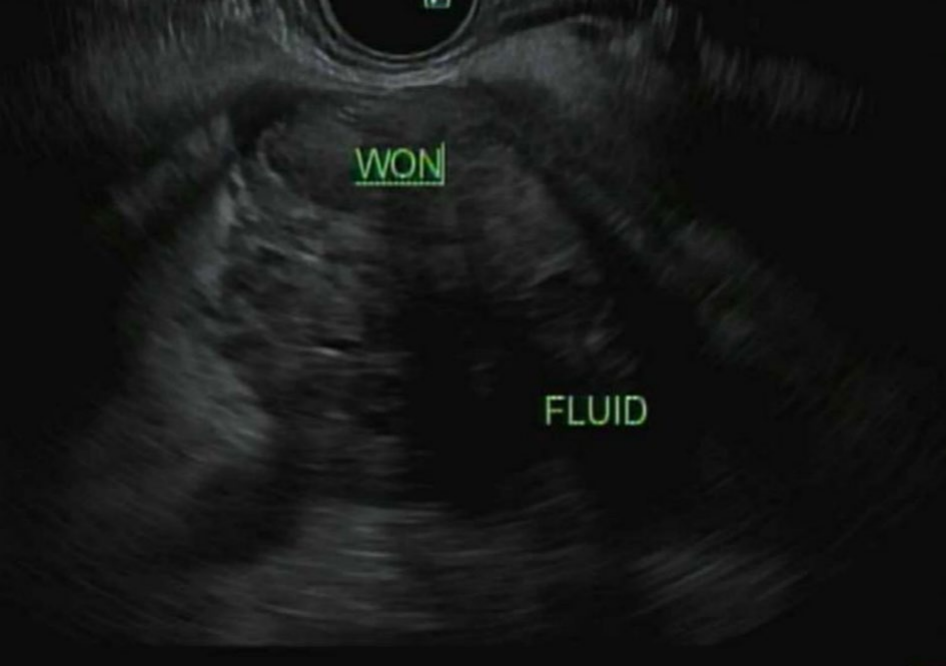
Initial EUS demonstrating walled-off necrosis in pancreatic tail

76-year-old male with chronic constipation was evaluated in the emergency department for abdominal and back pain and diagnosed with acute pancreatitis. He had no past medical history or risk factors for pancreatitis. The patient did not consume alcohol although he smoked cigars occasionally. A computed tomography (CT scan) of the abdomen and pelvis was significant for the presence of gallstones and peripancreatic fat stranding (Fig. 5). An MRI of the abdomen showed phlegmonous changes of the pancreatic body and tail with peripancreatic fluid extending to splenic hilum, probably a thrombus of splenic vein, and extensive peri-gastric and peri-splenic varices (Fig. 6). A questionable 7 mm enhancing lesion in the anterior left hepatic lobe, as well as multiple benign-appearing hepatic cysts and cholelithiasis were also seen. 2 months later, a CT abdomen showed progression of phlegmonous changes surrounding the pancreatic body and tail and progression of multiple low-density structures was seen throughout the pancreatic body and tail, which could reflect cyst/abscess formation or necrosis (Fig. 7). A low-density questionably rim-enhancing lesion was also noted, measuring 1.1 cm. An endoscopic ultrasound (EUS) was performed to evaluate further given the persistence of imaging findings. Endosonographic findings were pancreatic parenchymal abnormalities consisting of hyperechoic strands and lobularity, consistent with pancreatitis in both the head and body. A solid/cystic mass was identified in the pancreatic body and separately in the pancreatic tail, appearance most consistent with walled-off necrosis (Fig. 8).

**Fig. 9:**
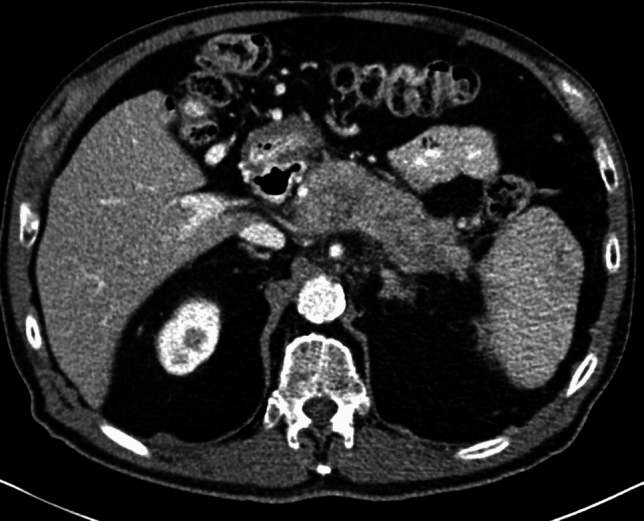
CT demonstrating hypoenhancing mass throughout the body and tail of the pancreas done at time of diagnosis of pancreatic cancer

**Fig. 10:**
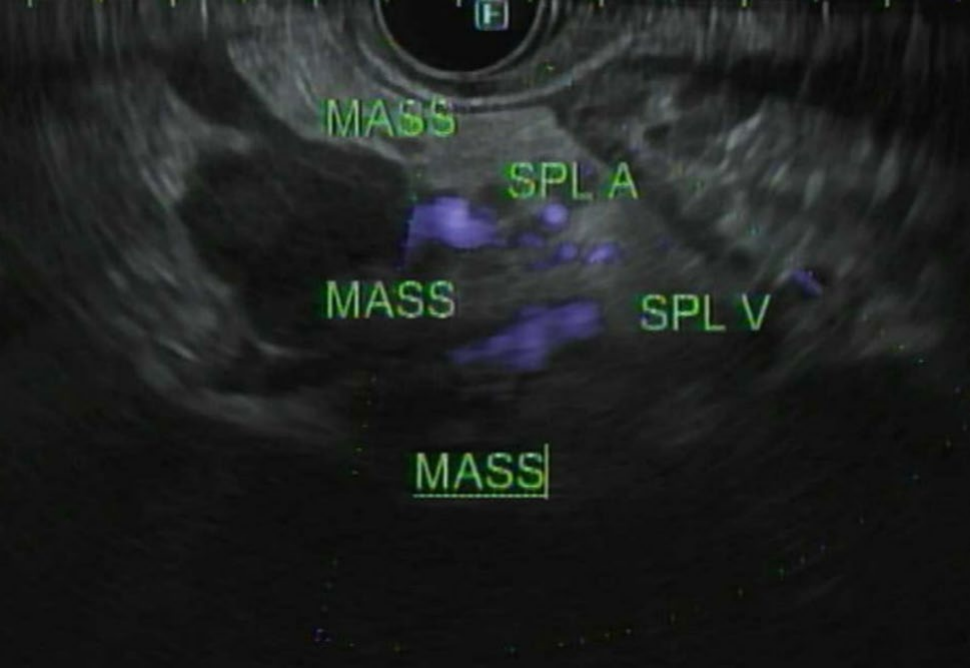
EUS done demonstrating mass in pancreatic tail

**Fig. 11:**
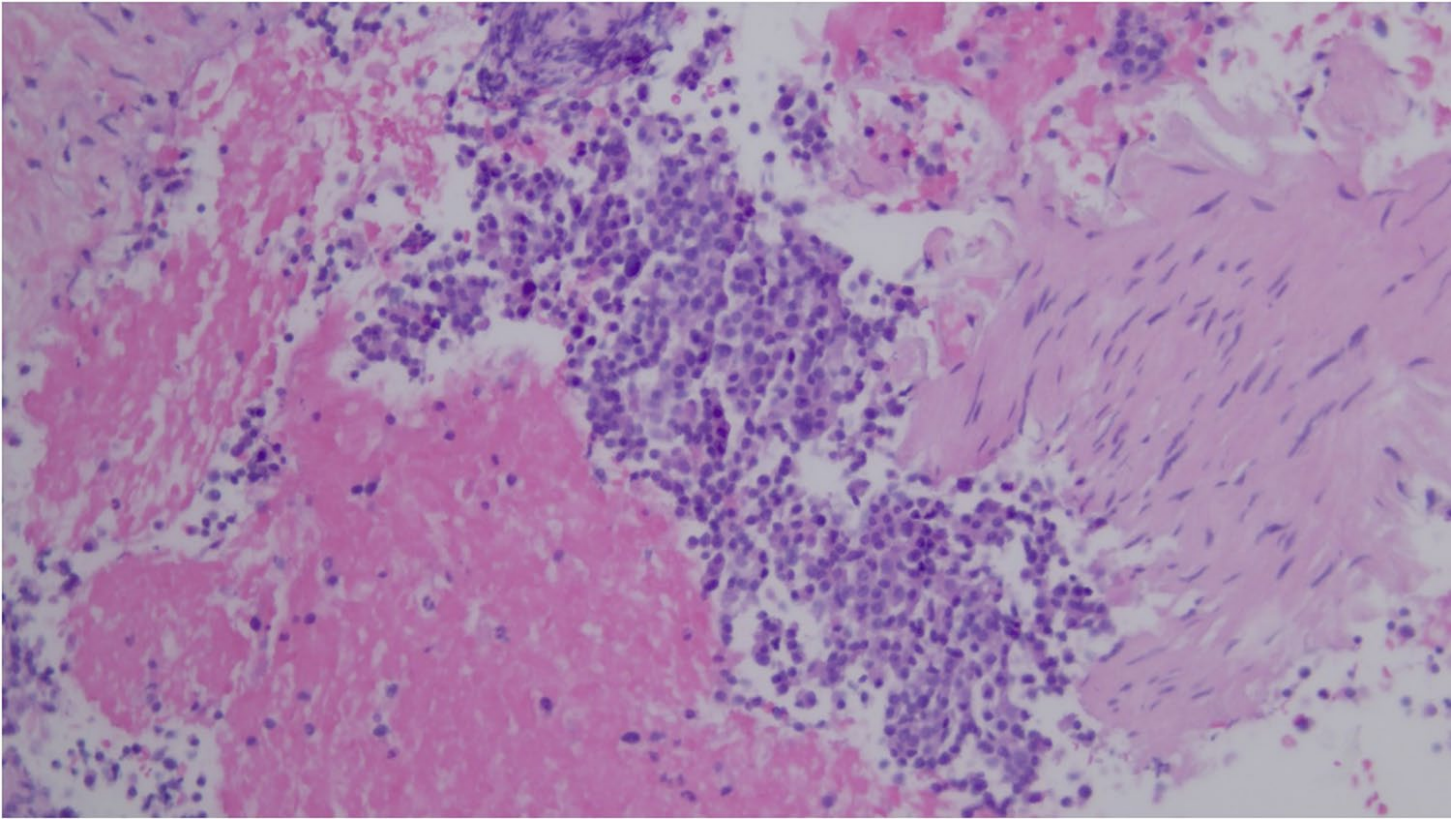
Pancreas FNA H&E 20 × magnification: Sheets of monotonous neoplastic cells with intermediate nuclear: cytoplasmic ratios, speckled nuclear chromatin, and eosinophilic cytoplasm. Necrosis present (not in pic). Immunostaining: positive for CAM 5.2 and synaptophysin

**Fig. 12:**
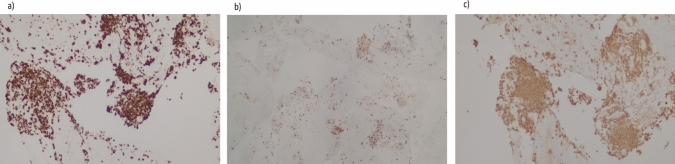
Showing Immunostaining stain X20 magnification, **a** CAM 5.2 keratin strongly positive on malignant cells, **b** Ki-67 labeling index: high labeling with 50% positive nuclei, **c** Synaptophysin immunostain: malignant cells are strongly positive

Given the risk of infection with needle puncture and since the patient was asymptomatic, the decision was made not to perform fine needle aspiration/biopsy. A repeat imaging was planned in 6 weeks. A CT abdomen/pelvis done for follow-up was then read as a known pancreatic body/tail mass with peripancreatic stranding concerning for a primary pancreatic neoplasm (Fig. 9). There were also two hepatic metastatic nodules, measuring up to 3.5 × 3.1 cm, representing worsening and disease progression. An EUS was repeated, and a 53 × 32 mm mass was identified in the pancreatic body with splenic artery and vein encasement (Fig. 10). A 38 × 28 mm mass in the left lobe of the liver was also seen (not previously identified). Biopsy was taken and histopathology was consistent with a well-differentiated neuroendocrine tumor, grade 3. The neoplastic cells expressed CAM 5.2, chromogranin, synaptophysin, lack dog 1, and Sox 10. Ki-67 reaching up to 40–50% in some foci (Figs. 11, 12). The case was discussed in the multidisciplinary tumor board and surgical resection was not recommended. PET/CT was done and evidence of tracer avid pancreatic neoplasm with tracer avid hepatic metastases (largest in the left lobe measuring ~ 4.7 cm with SUV max 46.0) and osseous metastatic lesions (one in the left ischium with SUV max 19.2). He was started on Octreotide + CAPTEM for metastatic NET and follow-up imaging after the first 3 months showed a partial response treatment.

## Discussion

Our case series highlights two patients diagnosed with pancreatic cancer (PC) following an episode of acute pancreatitis (AP). It is not uncommon for PC to be preceded by AP, with up to 5.9% of PC cases in the United States presenting similarly [2]. The mechanism behind this association is often attributed to pancreatic duct obstruction induced by the tumor, although various other correlations exist, contributing to the long-term impact of AP on PC formation [2, 7]. Generally, PC diagnosed after an AP episode carries a more favorable prognosis due to early stage detection and increased chances of surgical resection [2, 6]. However, our cases revealed diagnostic challenges that could potentially delay PC detection.

Following an episode of acute pancreatitis, as observed in our series, we noted peripancreatic fluid collection, which obscured the underlying tissue in conventional imaging. In addition, residual inflammation can limit the assessment of pancreatic tissue [8]. While it is reasonable to await the resolution of inflammation before delving into the etiology of pancreatitis, the spontaneous resolution of peripancreatic fluid collection may be prolonged. This delay could potentially mask or hinder the diagnosis of malignancy, particularly in the case of small, poorly enhancing solid tumors [9].

Providers may encounter cysts after an episode of acute pancreatitis, prompting questions about whether the cyst is a true pancreatic cyst, cystic degeneration of a pancreatic tumor, or wall formation following acute peripancreatic fluid collection [10]. Thus, even when patients exhibit typical pseudocyst imaging criteria, malignancy cannot be definitively excluded. In a surgical review involving 122 patients treated surgically for a pancreatic pseudocyst, 5 of them revealed cancer during the operation [11], and in a retrospective review of pseudocysts, 44% received a final diagnosis of pancreatic cystic neoplastic growth [12].

The probability of developing PC following an episode of acute pancreatitis (AP) is highest among elderly individuals, those with new-onset diabetes, individuals lacking a history of alcohol or biliary etiology, and those experiencing new-onset chronic pancreatitis [2, 13]. The first case highlights an additional diagnostic challenge posed by chronic pancreatitis, as it presents similar clinical manifestations to PC, such as weight loss, abdominal pain, and jaundice. Moreover, imaging may be constrained due to calcifications and inflammatory fluid collections associated with chronic pancreatitis [14]. Given it is crucial for early diagnosis of pancreatic cancer after the onset of AP, the literature suggests scheduled imaging within 3 months after discharge, such as CT or EUS. A follow-up CT within 3 months after discharge is beneficial, especially for patients with higher severity scores (CTSI ≥ 3) [15]. An unexplained acute distal pancreatitis is associated with an increased frequency of subsequent pancreatic cancer particularly pancreatic ductal adenocarcinoma resulting from obstruction of pancreatic duct by the tumor itself, and hence, the suggestion for EUS-guided biopsy should be considered in these patients on follow-up [16].

In cases of diagnostic challenges for possible pancreatic neoplastic growth, the most advantageous method is performing endoscopic ultrasound (EUS) for close pancreatic examination and tissue acquisition. Further molecular and histological investigation on EUS-obtained samples can help clarify the diagnosis [14]. However, conflicting results may arise between imaging and tissue analysis, necessitating a high index of suspicion for a diagnosis and possibly repeated EUS, despite its invasive nature. On the contrary, contrast-enhanced EUS (CE-EUS) has been shown to be effective in the diagnosis of pancreatic tumors, including those with cystic degeneration. One such study demonstrated a significant improvement in diagnostic yield for focal pancreatic lesions, increasing accuracy from 64% with unenhanced EUS to 91% with CE-EUS. This is particularly more evident in differentiation of cystic lesions, in which CE-EUS had a diagnostic yield of 96% compared to 71% with unenhanced [17].

Cystic degeneration in neuroendocrine tumors in pancreas varies across different studies and although this can occur in patients with pancreatic NETs, it s uncommon. Across different studies, around 10–18% of patients with pancreatic neuroendocrine tumors are associated with cystic degeneration [18].

Although most cases of PC stem from ductal origins, it is important to note that pancreatic adenocarcinoma is not the sole malignancy linked to AP. Our second case illustrates that neuroendocrine neoplasms can infrequently manifest as AP and can pose a diagnostic challenge due to post-AP alterations [19].

In conclusion, our case underscores the complex challenges involved in managing patients with concurrent pancreatic fluid collections, pancreatitis, and pancreatic adenocarcinoma. First, the association between chronic pancreatitis and an increased risk of pancreatic adenocarcinoma is complicated by the potential masking effect of calcifications and fluid collections. Second, the interpretation of imaging findings and cyst fluid characteristics can present conflicting information, as exemplified in our patient’s case where typical benign pseudocyst indicators conflicted with elevated CEA levels suggesting a mucin-producing cyst. In addition, both chronic pancreatitis and pancreatic cancer contribute to malnutrition and weight loss, further complicating the clinical picture. Lastly, while endoscopic ultrasound (EUS) stands out as the most accurate imaging test for identifying pancreatic cancer and enables tissue diagnosis, its invasive nature limits the frequency of serial examinations. These multifaceted challenges underscore the need for a comprehensive and nuanced approach to the care of patients with such complex and overlapping conditions.
